# The Related Metabolic Diseases and Treatments of Obesity

**DOI:** 10.3390/healthcare10091616

**Published:** 2022-08-25

**Authors:** Ming Yang, Shuai Liu, Chunye Zhang

**Affiliations:** 1 Department of Surgery, University of Missouri, Columbia, MO 65212, USA; 2 The First Affiliated Hospital, Zhejiang University, Hangzhou 310006, China; 3 Christopher S. Bond Life Sciences Center, University of Missouri, Columbia, MO 65212, USA

**Keywords:** obesity, comorbidities, molecular signaling pathways, treatment, drugs, clinical trials

## Abstract

Obesity is a chronic disease characterized by the abnormal or excessive accumulation of body fat, affecting more than 1 billion people worldwide. Obesity is commonly associated with other metabolic disorders, such as type 2 diabetes, non-alcoholic fatty liver disease, cardiovascular diseases, chronic kidney disease, and cancers. Factors such as a sedentary lifestyle, overnutrition, socioeconomic status, and other environmental and genetic conditions can cause obesity. Many molecules and signaling pathways are involved in the pathogenesis of obesity, such as nuclear factor (NF)-κB, Toll-like receptors (TLRs), adhesion molecules, G protein-coupled receptors (GPCRs), programmed cell death 1 (PD-1)/programmed death-ligand 1 (PD-L1), and sirtuin 1 (SIRT1). Commonly used strategies of obesity management and treatment include exercise and dietary change or restriction for the early stage of obesity, bariatric surgery for server obesity, and Food and Drug Administration (FDA)-approved medicines such as semaglutide and liraglutide that can be used as monotherapy or as a synergistic treatment. In addition, psychological management, especially for patients with obesity and distress, is a good option. Gut microbiota plays an important role in obesity and its comorbidities, and gut microbial reprogramming by fecal microbiota transplantation (FMT), probiotics, prebiotics, or synbiotics shows promising potential in obesity and metabolic syndrome. Many clinical trials are ongoing to evaluate the therapeutic effects of different treatments. Currently, prevention and early treatment of obesity are the best options to prevent its progression to many comorbidities.

## 1. Introduction

Obesity is a chronic disease characterized by the abnormal or excessive accumulation of body fat. A body mass index (BMI) that is calculated by the weight in kilograms divided by the square of the height in meters (kg/m^2^) is commonly used to define obesity. BMI < 25 kg/m^2^ is considered underweight or normal weight, followed by overweight (BMI = 25 to <30 kg/m^2^), moderate obesity (BMI = 30 to <35 kg/m^2^), and severe obesity (BMI ≥ 35 kg/m^2^) [1]. According to data from the World Health Organization (WHO), more than 1 billion people in the world are obese, including 650 million adults, 340 million adolescents, and 39 million children, which will cause about 167 million people to become ill by 2025 [2]. The prevalence of obesity is impacted by many genetic and environmental factors, such as sex, race, physical activity, diet, and socioeconomic status [3]. For example, a Japanese study shows that the prevalence of obesity in men is 27.2%, higher than that in women (10.6%) [4]. In addition, the personal or social background, including marital status, welfare enrollment, and current economic condition, is associated with obesity status in women, but not in men [4]. 

Many conditions can cause obesity, including a sedentary lifestyle, overnutrition, socioeconomic status, environment, and genetic factors [5]. For example, the COVID-19 pandemic contributed to an increased rate of obesity due to increased sedentary time and food consumption, and lack of socioeconomic activity [6]. Genes encoding leptin (LEP), leptin receptor (LEPR), melanocortin 4 receptor (MC4R), proprotein convertase subtilisin/kexin type 1 (PCSK1), proopiomelanocortin (POMC), kinase suppressor of ras 2 (KSR2), adenylate cyclase 3 (ADCY3), and others contribute to the development and progression of obesity [7]. For example, mutations of the *ADCY3* gene cause obesity in children from consanguineous Pakistani families, while heterozygous mutations are associated with the severity of obesity in children of European–American descent [8]. In addition, the *ADCY3* variant is associated with a significantly increased risk of obesity and T2D in the Greenlandic population [9]. *ADCY3* mutations play a pivotal role in neuronal primary cilia (microtubule-based cellular organelles) in neuronal function, which causes a predisposition to obesity [10]. 

Obesity is commonly associated with many other metabolic disorders, including type 2 diabetes (T2D), non-alcoholic fatty liver disease (NAFLD), cardiovascular diseases (CVDs), chronic kidney diseases (CKDs), and cancers [11,12]. In addition, obesity is positively associated with the severity and mortality of the coronavirus disease 2019 (COVID-19) in patients [13]. Adipose tissues secrete many inflammatory cytokines such as tumor necrosis factor α (TNF-α) and interleukin 6 (IL-6), which are a group of major contributing factors to metabolic disorders [14]. Obesity also causes other complications, such as dysfunction of vascular epithelial cells and lipid accumulation in organs except for adipose tissues. In the following sections, many factors that contribute to obesity-associated comorbidities are first reviewed. Then, molecular signaling pathways that are involved in the pathogenesis of obesity are discussed. Finally, current management and treatment options for obesity are summarized. 

## 2. Obesity, Its Comorbidities, and Associated Factors

Obesity, directly and indirectly, contributes to many other chronic disorders, including CKD, CVD, NAFLD, and T2D, as well as cancers, such as hepatocellular carcinoma (HCC) [15]. For example, the prevalence of NAFLD in obese patients can be as high as 70–90%, and is positively associated with BMI (≥35) [16]. Inflammation, insulin resistance, and metabolic dysfunction caused by obesity impact the mortality and morbidity of these chronic diseases. In this section, we review some common obesity-associated comorbidities and their associated factors. 

### 2.1. CKD

CKD is a leading public health problem worldwide, affecting about 13.4% of the world’s population. The most common symptoms in patients with CKD include sleep disturbances, weakness, fatigue, pain, and itchy skin [17]. The progression of CKD can lead to end-stage kidney disease, leading to an increase in renal replacement [18]. Obesity contributes to CKD by increasing intrarenal fat deposition, impaired glomerular filtration rate, and albuminuria [19]. In addition, obesity-associated local and systemic inflammation, insulin resistance, fibrogenesis, and gut microbiota dysbiosis are also associated with CKD development and progression [20,21,22]. Overweight and obese persons with metabolic disorders are more likely to develop CKD [23]. Thus, body weight control and a healthy diet are recommended for obese people. 

### 2.2. CVD

Obesity-associated factors including dyslipidemia, hypertension, insulin resistance, vascular endothelium dysfunction, and sleep disorders can contribute to CVD [24,25]. Obesity-associated comorbidities such as CKD and NAFLD also contribute to CVD [26,27]. For example, obesity-associated, low-grade chronic inflammation in metabolic tissues (e.g., adipose tissues and liver) alters the expression of adipocytokines and lipoproteins such as adiponectin and high-density lipoproteins (HDLs), which influences energy metabolism and causes endothelial dysfunction to increase the risk of CVD [28]. In addition, adipose tissues can secrete many other adipocytokines, such as leptin, resistin, visfatin, TNF-α, and IL-6 [29]. Their roles vary in the pathogenesis of CVD. Adipocytokines such as omentin and adiponectin, which are secreted from visceral adipose tissues (VATs), have anti-inflammatory functions. They can regulate the production of nitric oxide (NO) in endothelial cells and inhibit vascular calcification to prevent atherogenesis and inflammation [30]. In contrast, the expression of resistin and TNF-α contributes to insulin resistance in obesity and T2D. IL-6 is an important cytokine in myocardial lipid accumulation [29]. Blocking the expression of IL-6 or its receptor to the perturb IL-6 signaling pathway results in a reduction in the risk of coronary artery disease and atrial fibrillation, as well as T2D [31]. 

### 2.3. NAFLD

NAFLD is the most common type of chronic liver disease, affecting more than 25% of the world population [32,33]. NAFLD is a risk factor for T2D, CVD, and HCC. A large cohort study shows that being overweight and obese are positively and strongly associated with the prevalence of NALFD in metabolically healthy men and women, with multivariable-adjusted average hazard ratios of 2.15 and 3.55, respectively [34]. The prevalence of NAFLD and NASH will increase in multiple countries, as predicted in a model based on the prevalence of obesity and T2D [35]. Many obesity-related factors can contribute to NAFLD and its progression to non-alcoholic steatohepatitis (NASH), including dysfunction of subcutaneous white adipose tissues (scWAT) [36], insulin resistance [37], inflammation [38], dysbiosis of gut microbiota [39], and imbalance in energy metabolism [40]. 

Although obesity is a contributor to NAFLD, lean NAFLD patients also have a high risk of liver-related death compared to obese and overweight subjects [41]. These lean NAFLD patients do not have visceral fat accumulation (abdominal fat), with less fibrosis and a lower prevalence of T2D compared to obese patients, but commonly have dyslipidemia [42]. 

### 2.4. T2D

T2D is a chronic disease with a high level of blood glucose, contributing to the most common type of diabetes. In the comparison of insulin deficiency as a result of pancreatic β-cell damage in type 1 diabetes (T1D), insulin resistance is characterized as the major pathophysiological feature of T2D [43]. T2D affects more than 21.7 million people worldwide, leading to 1.5 million deaths in 2019 [44]. Factors such as physical activity, smoking, diet, and genetic conditions can lead to T2D development and progression. Obesity and insulin resistance can induce pancreatic islet hypertrophy and both α- and β-cell remodeling, disarray, and apoptosis, which can be ameliorated by fasting and anti-T2D treatments [45,46]. 

### 2.5. Cancers

Obesity has been identified to promote most cancer progression, including breast cancer [47,48], HCC [49,50], and pancreatic ductal adenocarcinoma (PDAC) [51,52]. Insulin resistance, adipose inflammation, and tumor-promoting growth factors such as fibroblast growth factor 1 (FGF1) are closely associated with most cancer progression [47]. In addition, obesity can impair anti-tumor immunity. For example, obesity-induced hepatic cholesterol accumulation selectively represses the anti-tumor effects of natural killer T (NKT) cells during diet-induced, NAFLD-related HCC [50]. A molecular study further shows that sterol regulatory element-binding protein 2 (SREBP2)-mediated excessive accumulation of cholesterol in hepatocytes causes lipid peroxide accumulation in NKT cells to reduce their cytotoxicity to tumor cells [50].

**Figure 1:**
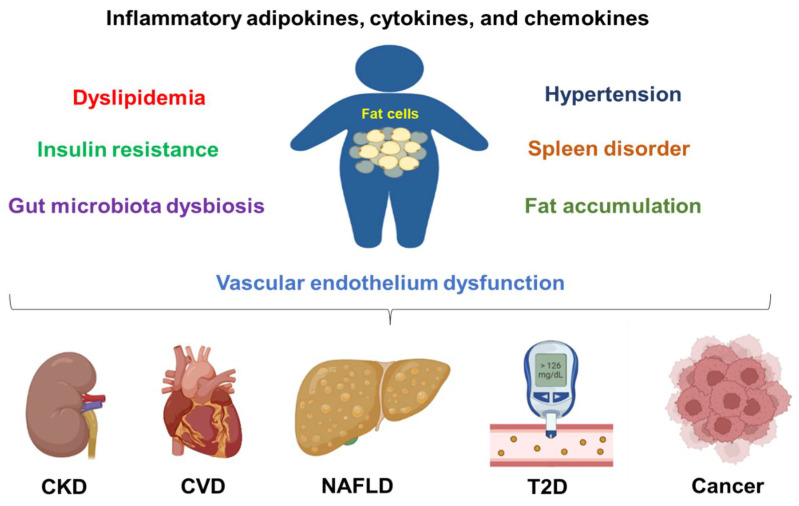
Obesity contributes to chronic disease development and progression. Obesity can cause inflammation, dyslipidemia, insulin resistance, hypertension, fat accumulation, vascular endothelium dysfunction, spleen disorder, and gut microbiota dysbiosis. All these conditions are contributing factors to chronic kidney disease (CKD), cardiovascular diseases (CVDs), non-alcoholic fatty liver disease (NAFLD), type 2 diabetes (T2D), and cancers.

Overall, obesity can induce chronic inflammation, metabolic syndrome, gut microbiota dysbiosis, and vascular epithelial cell dysfunction to promote the above-mentioned disease progression (Figure 1). Furthermore, obesity also contributes to other diseases, such as COVID-19 infection. One study shows that obesity increases the hospitalization and death rates in children and adolescents who were infected with COVID-19 [53]. The death rate increases in obese patients with a BMI over 40 kg/m^2^ [54].

## 3. Molecular Targets for Obesity Treatment

### 3.1. Inflammation-Associated Molecules

Elevated serum levels of inflammation markers, such as C-reactive protein (CRP), IL-6, TNF-α, and resistin are commonly found in patients with obesity and who are overweight [55,56,57], accompanying insulin resistance and other metabolic disorders. In contrast, a decreased expression of adiponectin is also found in the same patients. Adipose tissue inflammation is a major pathogenic feature of obesity. The inflammation is contributed to by adipokines, cytokines, and chemokines in adipose tissues, which are secreted from dysfunctional adipocytes and infiltrating immune cells [58]. For example, resistin can be secreted by adipocytes during adipogenesis to cause insulin resistance [57].

Increased vascular permeability and infiltration of leukocytes play an important role in obesity-induced systemic inflammation. For example, the administration of GW311616A, an inhibitor of neutrophil elastase, significantly attenuates vascular leakage, leukocyte infiltration, and the expression of proinflammatory cytokines in the white adipose tissues (WAT) in diet-induced obese mice [59]. The expression of adhesion molecules, such as intercellular adhesion molecule 1 (ICAM-1) and vascular cell adhesion molecule 1 (VCAM-1), is associated with the expression of adipogenesis and inflammation markers such as IL-6 and TNF-α [60].

The expression of low molecular weight (15–40 kDa) hyaluronan (HA) molecules is upregulated in the circulating blood of obese individuals, which causes an increased expression of pro-inflammatory cytokines such as IL-1β, IL-8, and monocyte chemoattractant protein-1 (MCP-1, or CCL2) in peripheral blood monocytes [61]. The underlying molecular mechanism study shows that those HAs induce the activation of nuclear factor kappa B (NF-κB) signaling, by regulating the phosphorylation of the α/β complex IκB kinase (IKK). 

### 3.2. Metabolic Syndrome-Associated Molecules

Metabolic syndrome (MetS) is characterized by abdominal obesity, dyslipidemia, hypertension, and insulin resistance [62,63]. Sirtuin 1 (SIRT1), a key metabolic regulator, plays a pivotal role in inflammation and lipid accumulation [64]. SIRT1 regulates the expression of sterol regulatory element-binding transcription factor 1c (SREBF1c) and peroxisome proliferator-activated receptor alpha (PPAR-α) in adipocytes, which play an essential role in the regulation of mitochondrial function [65]. In addition, the expression of molecules including leptin, adiponectin, and matrix metalloproteinases is regulated by SIRT1-mediated signaling pathways [65]. Upregulating the expression of SIRT1 in WAT in mice with high-fat diet (HFD)-induced obesity can ameliorate weight gain [66]. Meanwhile, SIRT1 activation plays a critical role in the inhibition of inflammation, oxidative stress, and cell apoptosis during diseases including obesity, diabetes, and CVDs. For example, an increased expression of SIRT1 in cardiomyocytes is associated with an improvement in insulin sensitivity [67]. 

### 3.3. Fatty Acid Metabolism-Associated Molecules

Fatty acid metabolism plays an important role in disease progression, including obesity-associated tumors. Monounsaturated fatty acids can bind G protein-coupled receptors (e.g., GPR120) and peroxisome proliferator-activated receptors (PPARs) to display anti-inflammatory effects by inhibiting macrophage M1 polarization and activating NF-κB and NLR family pyrin domain containing 3 (NLRP3) [68]. In contrast, the intake of medium-chain saturated fatty acids (MCSFAs), but not total saturated fatty acids (TSFAs), is associated with the risk of obesity or being overweight in Chinese adults, which is found in a study with a median of 11 years of follow-up [69]. A clinical trial (NCT02211612) also shows that the consumption of SFAs causes liver fat accumulation and increased serum levels of ceramides, whereas the consumption of polyunsaturated fatty acids (PUFAs) reduces the levels of ceramides, hyperlipidemia, and liver fat accumulation after excessive energy intake in obese persons [70]. 

### 3.4. Immune Checkpoints

The expression of programmed death-ligand 1 (PD-L1) in adipose tissues is found to be positively associated with visceral fat accumulation in obese or overweight persons. Conditional knockout PD-L1 on dendritic cells (DCs) increases weight gain and accelerates dietary obesity in mice [71]. Another study shows that the expression of PD-L1 in WAT in mice is associated with the expression of its receptor programmed cell death 1 (PD-1) in visceral WAT [72]. An increased PD-1 expression is found in type 2 innate lymphoid cells in adipose tissues to impair tissue metabolism, which is associated with the recruitment and activation of PD-L1^high^ M1 macrophages [73].

### 3.5. Exercise-Associated Signaling Molecules

A clinical study (NCT02753231) shows that a low-to-moderate intensity physical education class (LIPE), or its combination with a high-intensity physical education class, significantly regulates the expression of inflammatory or immune regulatory markers, including C-X-C motif chemokine ligand 13 (CXCL13, or BLC), C-C motif chemokine ligand 5 (CCL5), CCL11, CCL13, CCL18, and FGF6 [74]. A mouse study also shows that exercise reduces the expression of TNF-α and macrophage marker F4/80 in adipose tissues in HFD-fed mice, but it increases the expression of the M2 macrophage marker CD163, indicating phenotypic switching from M1 to M2 macrophages in adipose tissues [75]. Exercise can protect the obesity-induced mitochondrial dysfunction and oxidative stress [76], by altering specific lipid profiles including acylcarnitine, diacylglycerol, ceramide, and cardiolipins [77]. 

### 3.6. Toll-like Receptors

Toll-like receptors (TLRs) play essential roles in immunity, especially in the innate immune response, by recognizing pathogen-associated molecular patterns (PAMPs) from different microbiomes [78]. Some variants of the TLR-2 gene in exons 3 and 4 are reported to be significantly associated with the development of obesity in overweight persons [79]. TLR4 knockout inhibits HFD-induced obesity in young mice (≤6 months); however, TLR4^-/-^ mice develop spontaneous obesity with chronic low-grade inflammation in aged mice (18 months) [80]. Another study also shows that TLR4^-/-^ mice with HFD display a decrease in M1/M2 macrophage ratio, chronic inflammation, oxidative stress, and insulin resistance, with a higher subcutaneous fat/visceral fat ratio [81]. These changes are associated with the reprogramming of mitochondrial metabolism in adipose tissues. Furthermore, TLRs such as TLR4 and TLR9 contribute to obesity-associated metabolic disorders [82,83].

### 3.7. Intestinal Barrier-Associated Molecules

Gut microbiota plays a critical role in obesity [84,85]. Leakage of the gut barrier is commonly found during gut microbiota dysbiosis. Some gut microbial species such as a mucin-degrading bacterium *Akkermansia muciniphila* are involved in the protection of permeabilization of the gut barrier and systemic inflammation [86]. These bacteria also contribute to intestinal stem cell-mediated epithelial cell development and a decreased population of B cells in the intestine [86,87]. In addition, treatment of *A. muciniphila* can decrease serum levels of inflammatory cytokines (e.g., IL-2 and IFN-γ) and lipid overload, and increase fatty acid oxidation in adipocytes [88]. 

**Figure 2:**
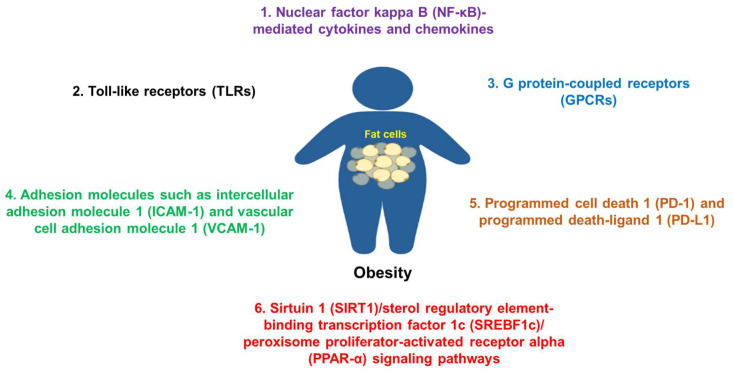
Some common molecules and their signaling pathways in the pathogenesis of obesity. Molecules such as NF-κB, TLRs, adhesion molecules, GPCRs, PD-1/PD-L1, SIRT1, and others are commonly involved in the pathogenesis of obesity.

Overall, many molecules or molecular signaling pathways are involved in the pathogenesis of obesity and its commodities (Figure 2), which are good targets for potential therapies.

## 4. Clinical Management of Obesity

**Figure 3:**
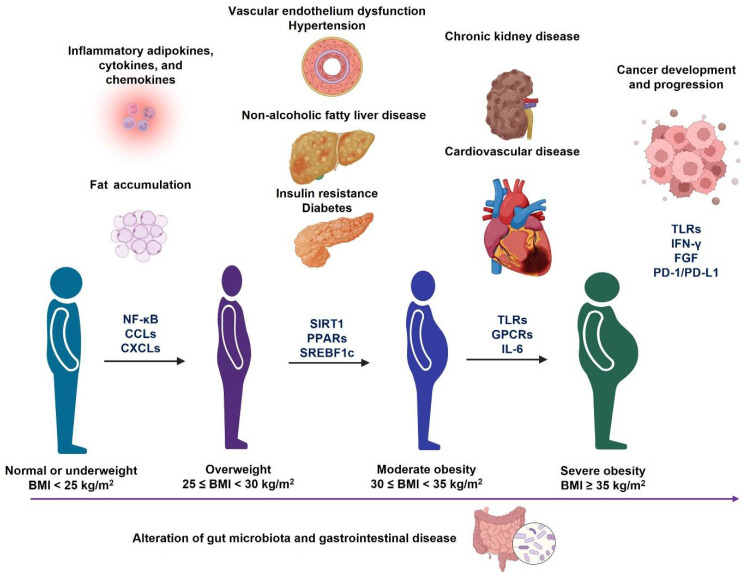
Different metabolic disorders accompany the development and progression of obesity. During the progression of obesity from overweight to severe obesity, it may cause different metabolic disorders, which are associated with the alteration of gut microbiota profiles. In addition, the molecular signaling pathways (Figure 2) may also change during the progression of each obesity-associated comorbidity.

**Figure 4:**
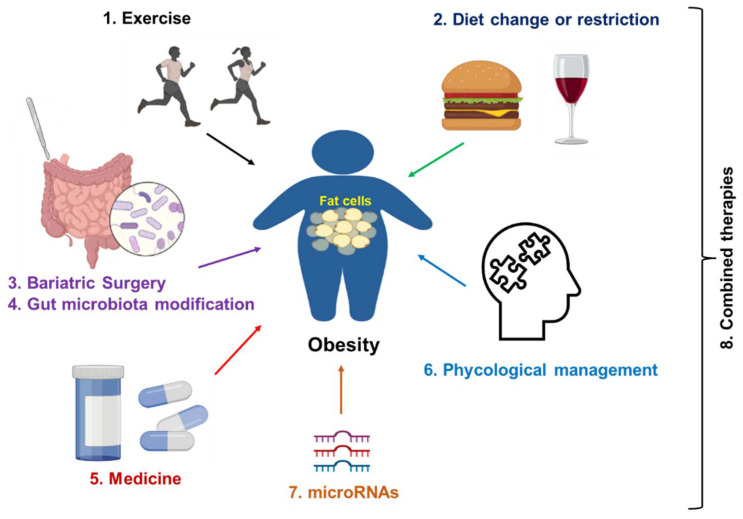
Strategies for obesity treatment. Exercise and dietary change or restriction are commonly applied to prevent and treat the early stage of obesity. Bariatric surgery is a commonly applied treatment for server obesity. Medicines such as semaglutide and liraglutide can be used as monotherapy or a synergistic treatment together with lifestyle management. In addition, psychological management, especially for patients with obesity and distress, is a good option. Gene regulation (microRNAs-mediated therapies) will be applied in the future. Finally, a combination of two therapies or multiple treatments from therapies 1 to 7 can be applied to improve the treatment efficacy.

Obesity is a major contributing factor to many diseases at different stages of obesity (Figure 3); therefore, it is critically important to prevent and treat obesity. Here, we review some commonly applied strategies in obesity prevention and treatment (Figure 4). In addition, the underlying mechanism of each treatment is also discussed.

### 4.1. Exercise

A meta-analysis study shows that exercise, especially aerobic exercise, significantly decreases the N-terminal-pro hormone B-type natriuretic peptide (BNP) in patients with heart failure, regardless of obesity status [89]. A 12 week exercise program combining aerobic and resistance training on obese β-amyloid-treated rats significantly alleviated the side effects of β-amyloid plaque on brain function, and improved cardiopulmonary function, muscular endurance, and short-term memory ability [90]. This exercise training also activated the expression of many proteins that are associated with brain function in the hippocampus and cerebral cortex, including peroxisome proliferator-activated receptor gamma coactivator 1-alpha (PGC-1α), fibronectin type III domain-containing protein 5 (FNDC5), and brain-derived neurotrophic factor (BDNF). 

### 4.2. Diets

#### 4.2.1. Carbohydrate-Restricted Diets

High-carbohydrate diets usually contain carbohydrates accounting for more than 45% of total calories [91]. Carbohydrate-restricted diets can be classified as moderately low carbohydrate diets (MCD), low carbohydrate diets (LCD), and very low carbohydrate diets (VLCD), with carbohydrates accounting for 26–45%, 10–25%, and less than 10% of total caloric intake, respectively. The MCD and LCD are recommended for adults with obesity or who are overweight as a dietary regimen for weight loss in South Korea [92]. A recent clinical trial study (NCT03814694) shows that weight reduction induced by moderate dietary carbohydrate restriction has no clinically important impact on health-related quality of life and global cognition in obese or overweight patients with T2D [93]. 

#### 4.2.2. Low-Fat Diet

Treatment with a low-fat diet (LFD) in obese patients reduces the total cholesterol (TC) but not triglycerides (TG). LFD plus moderate-intensity aerobic exercise training (MIAET) significantly decreases both TC and TG and improves depression status, showing greater advantages compared to single treatment alone [94]. 

#### 4.2.3. Fiber Diet

Dietary fiber, a complex mixture of molecules mainly including polysaccharides, plays a pivotal role in the reduction in obesity and its comorbidities [95]. The underlying mechanisms show that the consumption of a high fiber diet can alter gut hormone secretion (e.g., glucagon-like peptide-1 or GLP-1 and peptide YY or PYY), appetite, secondary bile acid production, energy metabolism, and insulin sensitivity. Twelve week consumption of rye products, a high fiber product with whole grains, causes a greater weight loss and body fat loss, as well as a reduction in C-reactive protein, than the wheat group [96].

#### 4.2.4. Mediterranean Diet

The Mediterranean diet (MedDiet), which consists of unsaturated fats, whole grains, fruits and vegetables, fish, nuts, and legumes, displays the function to reduce bodyweight, and improve obesity and its comorbidities [97,98,99]. For example, a clinical study (NCT04845373) compares the effect of MedDiet with LFD on obese adolescents with NAFLD. MedDiet consists of target macronutrient energy, with 40% from carbohydrates, 35–40% from fats (with <10% of energy as saturated fats), and 20% of energy as proteins, as well as consumption of fish and legumes at least 2–3 times a week, and walnuts and olive oil every day. LFD is composed of target macronutrient energy, with 50–60% from carbohydrates, <30% from fats (with <10% of energy as saturated fat), and 20% from proteins [99]. After a 12 week consumption of MedDiet and LFD, both groups show a significant decrease in hepatic steatosis, serum transaminase levels, and insulin resistance. Subjects in the MedDiet group have lower levels of aspartate aminotransferase (AST) and increased total serum antioxidant ability, with increased levels of paraoxanase-1 and glutathione peroxidase compared to those in the LFD group (*p* < 0.05). In addition, the MedDiet decreases the CRP levels, while LFD treatment decreases IL-6 compared to basal levels. Another meta-analysis also shows that MedDiet adherence is negatively associated with overweight and/or obesity risk and 5 year weight gain in adults [100]. Some of these examples are listed in Table 1. Overall, appropriate energy limitation and the consumption of low-fat and high-fiber diets improve obesity-related metabolic disorders.

### 4.3. Bariatric Surgery

Bariatric surgery (BS) is shown to be successful in the treatment of obesity and its comorbidities and the improvement of the quality of life in patients with severe obesity (BMI ≥ 35) [101]. The weight loss outcomes such as total weight loss and final BMI impact the quality of life after bariatric surgery [102]. Roux-en-Y gastric bypass (RYGB) and sleeve gastrectomy (SG) are the two most commonly used BS procedures for the treatment of obesity and its comorbidities [103]. For example, laparoscopic gastric bypass or SG surgeries significantly decrease BMIs and improve blood glucose levels postprandial in overweight or obese patients with T2D [104]. Some studies show that RYGB is more effective in weight loss than SG in the treatment of obese patients with T2D, but the T2D remission rates are similar post both procedures [105,106,107]. 

The underlying mechanisms of BS in the improvement of obesity and its comorbidities include the reduction of energy absorption, increase in gut hormone secretion, and improvement of gut microbiota balance. Both RYGB and SG treatments can alter gut hormones to achieve weight loss, such as PYY, GLP-1, and ghrelin. A study shows that RYGB increases blood levels of PYY and GLP-1 at both 26 and 52 weeks after surgery compared to baseline, while SG increases PYY and GLP-1 at 26 weeks, but not at 52 weeks, and decreases blood ghrelin levels in patients compared to baseline [108]. Different levels of these gut hormones induced by BS procedures may have different effects on weight loss. An analysis of gut microbiota profiles in obese patients after bariatric surgery shows that the abundance of genus *Blautia* is decreased and the abundance of genus *Bacteroides* is increased after BS, and the ratio of *Blautia*/*Bacteroides* is positively associated with BMI [109]. Overall, BS can restrict or reduce energy absorption, increase the secretion of gut hormones, and balance the gut microbiota profile to treat obesity.

### 4.4. Medicines

Currently, there are anti-obesity medications approved by the Food and Drug Administration (FDA) in the United States, including (1) noradrenergic agonists phentermine, benzphetamine, and phendimetrazine, (2) a serotonin (SE)–norepinephrine (NE)–dopamine (DA)-releasing agent diethylpropion, (3) an opioid receptor antagonist naltrexone with a DA and NE reuptake inhibitor bupropion, (4) a selective 5-hydroxytryptamine 2C (5HT-2C) receptor agonist lorcaserin, (5) a gastrointestinal and pancreatic lipase inhibitor orlistat, (6) GLP-1 analog liraglutide, (7) a GLP-1 receptor agonist semaglutide, and (8) a melanocortin-4 receptor (MC4R) agonist setmelanotide, as well as (9) phentermine with a gamma-aminobutyric acid (GABA) agonist topiramate extended-release [110,111]. The underlying mechanisms of these molecules were reviewed in a previous paper [111]. In addition, some of these are less prescribed by doctors, or have been withdrawn (e.g., diethylpropion in Europe) due to their side effects. Many treatment options are currently under clinical trial evaluation. For example, one single-blinded study shows that a subcutaneous infusion of hormones including PYY, GLP-1, and oxyntomodulin (OXM) at a dose of 4/4/0.4 pmol/kg/min for 4 weeks significantly reduces body weight and improves glucose tolerance in patients with obesity and diabetes [112]. Here, we review some of these treatments in clinical trials (Table 2). 

### 4.5. Management of Gut Microbiota

Gut microbiota plays an important role in many metabolic disorders [15,124,125], including obesity. Mechanistic studies show that gut microbiota can impact diet metabolism and energy balance, gut permeability, intestinal hormone secretion, insulin resistance, and systemic inflammation [126,127]. All these factors are associated with obesity-induced comorbidities. Many factors can cause an alteration of the gut microbiota profile to impact obesity, including diet [128], BS [109], physical activity [129,130], probiotics (e.g., *Bifidobacterium* and *Lacticaseibacillus*) [131,132], prebiotics, and synbiotics (containing both probiotic and prebiotic components). 

A single U.S. academic medical center clinical trial (NCT02530385) shows that weekly administration of fecal microbiota transplantation (FMT) capsules from healthy lean donors in adults with obesity for 12 weeks results in gut microbiota engraftment in most recipients, without causing adverse events [133]. Another single-center study (NCT02637115) shows that daily oral supplementation of either live or pasteurized *A. muciniphila* (10^10^ colony-forming units or CFUs) for three months is well-tolerated and safe [134]. Treatment with pasteurized *A. muciniphila* significantly improves insulin sensitivity and decreases plasma total cholesterol, and slightly decreases body weight (*p* = 0.091) compared to the placebo group. In addition, supplementation of *A. muciniphila* for three months decreases the expression of blood markers for liver dysfunction and inflammation without changing the gut microbiota profile [134]. A 12 week supplementation of probiotics *Lactobacillus curvatus* HY7601 and *Lactobacillus plantarum* KY1032 (10^10^ CFUs) significantly decreases body weight, visceral fat mass, and waist circumference, but increases the expression of adiponectin compared with the placebo group [135]. This treatment changes the gut microbiota profile, causing an increase in the abundance of the *Bifidobacteriaceae* and *Akkermansiaceae* families, and a decrease in the abundance of the *Prevotellaceae* and *Selenomonadaceae* families. Supplementation of synbiotics increases the abundance of beneficial bacteria *Bifidobacterium* and *Lactobacillus* in the gut. There is a negative association between overtime blood glucose levels and the abundance of *Lactobacillus*, but there is a positive association between the abundance of *Bifidobacterium* and overtime obesity features including body mass, BMI, waist circumstance, and body fat mass [136]. 

Additionally, the common BS procedures RYGB and SG can reduce body weight and improve obesity-associated inflammation and insulin resistance by altering gut microbiota (e.g., reduction in genera *Butyriciccocus*, *Eubacterium ventriosum*, and *Monoglobus*) [137]. Another study shows that the consumption of high-fiber rye foods causes an increase in the abundance of butyrate-producing *Agathobacter,* and a reduction in the abundance of *Ruminococcus*, which is associated with a reduction in low-grade inflammation and an increase in plasma butyrate [138]. Overall, current clinical studies show the promising effects of different gut microbial intervention strategies on obesity and its comorbidities. A large scale of clinical trials and different compositions of gut microbial species should be investigated in the future for evaluating the efficacy of gut-microbiota-mediated therapies against obesity. 

### 4.6. Psychological Management

Psychological interventions are shown to be effective in the treatment of emotional or mental disorders. Depression is also associated with the development of obesity and its comorbidities [139]. For example, a study in German adults shows that the prevalence of comorbid depression and obesity in men is 1.3% (95% confidence interval/CI: 0.8–2.0), which increases to 2.0% (95% CI: 1.3–3.0) in women [140]. Another cohort study reveals that middle-aged and older Chinese people (≥45 years) with comorbid depression and obesity have a higher risk of developing functional disability than those with depression or obesity alone and without both [141]. Therefore, psychological management may benefit the treatment of obesity.

### 4.7. MicroRNA-Mediated Therapy

MicroRNAs (miRNAs) are a class of small, non-coding RNAs that play important roles in host health and disease [142]. Recent studies also reveal that miRNAs are involved in adipogenesis and obesity [143], which are potential targets for obesity treatment. For example, miR-203 can target an apical sodium-dependent bile acid transporter to regulate bile acid homeostasis and decrease obesity and dyslipidemia [144]. In addition, the expression of circulating miRNAs such as miR-99-5p/100-5p can be regulated by lifestyle intervention, and is associated with a decrease in fat accumulation and an improvement in glucose metabolism in patients with abdominal obesity [145]. Furthermore, some microRNAs, such as miR-29 family members miR-29a-3p, miR-29b-3p, and miR-29c-3p, are associated with different metabolic disorders, including obesity, insulin resistance, and T2D; therefore, they are potential therapeutic and prognostic markers in obesity and obesity-related metabolic disorders [146,147]. Regulation of target miRNAs can be applied to treat obesity (e.g., miR-21 and miR-502-3p) [148,149] and its comorbidities such as NAFLD (e.g., miR-802 and miR-144) [150,151], T2D (e.g., miR-150 and miR-26a) [152,153], and CVD (e.g., miR-181b and miR-126-5p) [154,155].

## 5. Limitations and Future Perspectives

Under-recognition, low socioeconomic status, time constraints, and many comorbidities increase the difficulty of obesity treatment [156]. In addition, obesity is a relapsing disease. The recommended lifestyle intervention such as dietary change and physical activity may not be sufficient to maintain the bodyweight loss [157]. BS is a very effective strategy for patients with severe obesity [158]. However, the treatment cost, lack of care source, and concerns about the adverse effects limit the application of surgical procedures in the clinic. Moreover, insufficient delivery of anti-obesity medicines reduces their effectiveness, and brings potential side effects [159]. 

New diagnosis and prognosis markers should be developed to monitor disease development and progression. The levels of fasting plasma glucose (FPG) and fasting insulin (FI) can be applied to predict the success of weight loss in response to a low-carbohydrate or low-fat diet [160]. Increased FPG levels are associated with the success of weight loss and maintenance in overweight patients with consumption of diets with a low glycemic load or with large amounts of fiber and whole grains [161]. Meanwhile, technological advancements in wearable sensors help to monitor the treatment efficacy and help in the early detection of the risk of obesity and its comorbidities by measuring heart rate, respiratory rate, temperature, and blood pressure [162,163]. Wearable devices are also very useful tools for self-management and physical activity tracking [164].

The evaluation of new treatments before clinical trials are also required. Preclinical animal models have been broadly applied to study the underlying cellular and molecular mechanisms of obesity and to evaluate the treatment effectiveness of medicines. The murine models of obesity have been well-summarized in another paper [165].

## 6. Summary

Obesity is one the most common metabolic diseases, affecting about 13% of adults worldwide. In addition, 39 million children (≤5 years old) were overweight or obese in 2020, according to the data reported by the WHO on 9 June 2021. Obesity contributes to other metabolic disorders, such as T2D, NAFLD, CKD, CVD, and cancers. A sedentary lifestyle, overnutrition, socioeconomic status, environments, and genetic mutations are common factors that cause obesity. There are many molecular signaling pathways in the pathogenesis of obesity, including TLRs, NF-κB, GPCRs, and SIRT1. There are potential targets for obesity management. Current treatment options include exercise and dietary change or restriction for the early stage of obesity, bariatric surgery for severe obesity, and medicines such as semaglutide and liraglutide that can be used as monotherapy or a synergistic treatment with other treatments. In addition, psychological management, especially for patients with obesity and distress, is a good option. Many clinical trials are ongoing to evaluate the therapeutic effects of different treatments. Despite the progression, more efforts are required to search for novel therapeutic options, such as gene regulation (e.g., microRNAs) and gut microbiota reprogramming. Overall, a combined therapy (Figure 4) may be the most effective way to prevent and treat obesity and its comorbidities, due to the complex pathogenesis of this disease.

## Figures and Tables

**Table 1:** Dietary components and their effects on obesity.

Diets	Components	Effects	References
Carbohydrate-restricted diets	Moderately low carbohydrate diets (MCD) and low carbohydrate diets (LCD) contain carbohydrates accounting for 26–45% and 10–25% of total caloric intake, respectively.	Weight reduction	[92]
Carbohydrate-reduced high-protein diet	Energy components include 30% of carbohydrates, 30% of proteins, and 40% of fibers.	Weight loss	[93]
Low-fat diet	Target macronutrient energy includes 50–60% from carbohydrates, <30% from fats (with <10% of energy as saturated fats), and 20% from proteins.	Decrease in hepatic steatosis, serum transaminase levels, and insulin resistance	[99]
High-fiber diet	Consumption of high-fiber rye products as part of a hypocaloric diet for 12 weeks causes a greater weight loss and body fat loss, as well as a reduction in C-reactive protein, compared to refined wheat.	Weight loss and body fat loss, as well as a reduction in C-reactive protein	[96]
Mediterranean diet	Target macronutrient energy includes 40% from carbohydrates, 35–40% from fat (with <10% of energy as saturated fats), and 20% of energy as proteins, as well as consumption of fish and legumes at least 2–3 times a week and walnuts and olive oil every day.	Decrease in hepatic steatosis, serum transaminase levels, and insulin resistance	[99]

**Table 2:** Anti-obesity medicines in clinical trials.

Classes	Medicine	Clinical Trials	Phase	Outcomes	References
GLP-1 mimetics or analogs	Exenatide	NCT00856609	3	Subcutaneous treatment of exenatide 10 μg twice daily decreases early ad libitum energy intake.	[113]
	Semaglutide	NCT03552757	3	At week 68, 68.8% of adult patients with obesity or type 2 diabetes who received treatment of semaglutide 2.4 mg once a week achieve an average of 9.6% of bodyweight reduction compared to the baseline.	[114]
	Semaglutide	NCT03548987	3	Maintaining weekly treatment with subcutaneous 2.4 mg of semaglutide causes a continuous weight loss compared to placebo in adults who received a 20 week run-in period with subcutaneous semaglutide.	[115]
	Liraglutide	NCT03480022	3	Treatment with liraglutide 3 mg once daily reduces bodyweight and androgenicity, and improves cardiometabolic parameters in women with obesity and polycystic ovary syndrome compared to placebo.	[116]
GLP-1 agonist	Efpeglenatide	NCT02075281	2	Treatment with efpeglenatide 4 mg or 6 mg once weekly and 6 mg or 8 mg once every 2 weeks, significantly reduces body weight and improves glycemic control compared to placebo in patients with obesity or who are overweight with comorbidities and without T2D.	[117]
Lifestyle intervention and medicine	Semaglutide plus lifestyle intervention	NCT03548935	3	Treatment of 2.4 mg of semaglutide once weekly plus lifestyle intervention is associated with a sustained, clinically relevant reduction in body weight.	[118]
Lifestyle intervention and medicine	Semaglutide plus a low-calorie diet and intensive behavioral therapy	NCT03611582	3	Treatment with semaglutide alone (2.4 mg weekly) significantly reduces body weight in adults who are overweight or obese compared with placebo. Using this treatment as an adjunct to intensive behavioral therapy and an initial low-calorie diet in the first 8 weeks results in further weight loss within 68 weeks.	[119]
Lifestyle intervention and medicine	Liraglutide (3.0 mg) plus lifestyle therapy	NCT02918279	3	In adolescents with obesity, the use of liraglutide (3.0 mg) plus lifestyle therapy leads to a significantly greater reduction in the BMI standard deviation score than placebo plus lifestyle therapy.	[120]
Lifestyle intervention and medicine	A combining exercise and liraglutide therapy	NCT04122716	4	The combination strategy of exercise and liraglutide leads to greater weight loss compared to exercise alone, improving the glycated hemoglobin level, insulin sensitivity, and cardiorespiratory fitness.	[121]
Lifestyle intervention and medicine	Weight management program (WMP) plus one of the U.S. FDA-approved medicine	NCT03799198	4	The estimated percentage of participants with at least 5% weight loss is significantly increased in the group treated with WMP plus with one of five of the U.S. FDA-approved medications (orlistat, lorcaserin, phentermine/topiramate, naltrexone/bupropion, and liraglutide, 3.0 mg) medication (62.5%) than WMP alone (44.8%) (*p* = 0.02).	[122]
Decreasing appetite	VI-0521	NCT02714062	4	Treatment with VI-0521 (a fixed-dose combination of immediate-release phentermine and extended-release topiramate) in adolescents with obesity causes ≥5% of weight loss within day 56 at both low and high doses, which are safe and well-tolerated.	[123]

## Data Availability

Not applicable.
